# Asynchronous Stabilization for Two Classes of Stochastic Switching Systems with Applications on Servo Motors

**DOI:** 10.3390/e24081126

**Published:** 2022-08-15

**Authors:** Yushu Deng, Shihao Wang, Shiqi Zheng, Haiming Li, Haitao Jian, Xiaoqi Tang

**Affiliations:** 1 Shaoyang Institute of Advanced Manufacturing Technology, Shaoyang 422000, China; 2 School of Automation, China University of Geosciences, Wuhan 430074, China; 3 Hubei Key Laboratory of Advanced Control and Intelligent Automation for Complex Systems, Wuhan 430074, China; 4 Engineering Research Center of Intelligent Technology for Geo-Exploration, Ministry of Education, Wuhan 430074, China; 5 School of Mechanical Science and Technology, Huazhong University of Science and Technology, Wuhan 430074, China

**Keywords:** asynchronous stabilization, Markov jump system, stochastic switching systems, control system

## Abstract

This paper addresses the asynchronous stabilization problem of two typical stochastic switching systems, i.e., dual switching systems and semi-Markov jump systems. By dual switching, it means that the systems contain both deterministic and stochastic switching dynamics. New stability criteria are firstly proposed for these two switched systems, which can well handle the asynchronous phenomenon. The conditional expectation of Lyapunov functions is allowed to increase during some unmatched interval to reduce the conservatism. Next, we present numerically testable asynchronous controller design methods for the dual switching systems. The proposed method is suitable for the situation where the asynchronous modes come from both inaccurate mode detection and time varying delay. Meanwhile, the transition probabilities are both uncertain and partly accessible. Finally, novel asynchronous controller design methods are proposed for the semi-Markov jump systems. The sojourn time of the semi-Markov jump systems can have both lower and upper bounds, which could be more practical than previous scenarios. Examples are utilized to demonstrate the effectiveness of the proposed methods.

## 1. Introduction

Switched systems, as a special class of hybrid systems, have received considerable attention in the past few years [1,2]. It usually contains a family of subsystems and a switching law that coordinates between them [3]. A variety of physical systems, such as mechanical systems [4,5] and network control systems [6,7,8,9], can be well modeled by the switched systems. Due to the abrupt and unpredictable phenomenons in real practice, a stochastic switching law is usually adopted for the switched systems. Hence, research on stochastic switching systems are of great practical importance [10,11]. A large number of excellent results have been obtained for various aspects of stochastic switching systems, such as stability analysis [12,13], controller design [14,15,16,17], state estimation [18,19], etc.

Markov jump systems are a typical class of stochastic switching systems [20,21,22,23]. The stochastic switching law is described by a Markov process. Numerous works have been conducted on the Markov jump systems. For instance, Ref. [24] considered the problem of state feedback stabilization for singular Markov jump systems by using the equivalent sets technique. In [25], a sliding mode controller was designed for a Markov jump system with digital data transmission. More recently, Ref. [10] considered the stabilization problem of a class of Markov jump systems with generally uncertain transition rates. Namely, the transition probability is time-varying and governed by another deterministic switching law [26]. In fact, the system in [10] is a kind of *dual switching systems* [27]. By dual switching, it means that the systems contain both stochastic and deterministic switching law. Many practical systems can be described by dual switching systems. For example, consider a serve motor system. The motor may work in different situations, such as no load, external load, external inertia, etc. This can be represented by the deterministic switching. The fast time-varying parameters, such as from stochastic disturbance, abrupt failure, and noise, can be expressed as a stochastic switching sequence. Another application example is given by a multi-loop networked control system suffered from failures. The stochastic failures of the communication network can be expressed as a Markov chain, while the scheduling signal selects which control loop is currently attended. Due to this hybrid feature, the controller design problem becomes more difficult.

In contrast with Markov jump systems, semi-Markov jump systems, recently, have drawn increasing attention [28,29,30,31]. It can be dated back to the work in Howard [32]. Compared with Markov jump systems, the sojourn time for the semi-Markov jump systems can satisfy various kinds of probability distributions, such as geometric distribution, Weibull distribution, Bernoulli distribution etc. Hence, semi-Markov jump systems are able to represent a much more general class of real systems. However, this feature gives the transition probabilities in semi-Markov jump systems a “memory” property, which brings significant difficulties to the stability analysis and controller design. In the earlier works on semi-Markov jump systems, some special classes of probability distributions of sojourn time were considered [33]. Recently, by introducing the semi-Markov kernel, Ref. [34] analyzed the stability of semi-Markov jump linear systems. Then, an LMI-based design method was proposed to compute the stabilizing controller gain. More recently, Ref. [11] considered the stabilization issues of a family of semi-Markov jump systems with *both lower and upper bounds of sojourn time*. This kind of system is more general and practical than the previous works. In fact, by considering the lower bound of the sojourn time, less conservative results could be obtained for controller design.

In real engineering, the system modes information for the switched systems is often not fully accessible. This is the so-called asynchronous phenomena in the controller design [34,35,36,37]. This phenomena may be caused by the communication delay in the network or the missing measurement of the mode detector. Therefore, it is a challenge issue to design asynchronous controllers for the switched systems, especially stochastic switching systems. Ref. [34] proposed a novel asynchronous controller design method for deterministic switched systems with average dwell time. Ref. [38] addressed the asynchronous sliding mode control problem for delayed singular Markov jump systems. An asynchronous $H_{\infty }$ control method was recently proposed in [39] for 2D Markov jump systems in Roesser Model. However, few articles have paid attention to asynchronous stabilization of the dual switching or semi-Markov jump systems.

Motivated by the above thoughts, this paper will conduct a further study on the asynchronous stabilization problem of two typical stochastic switching systems, i.e., dual switching and semi-Markov jump systems. The contributions are in the following points:

1. New stability criteria are proposed for the considered stochastic switching systems, which can well handle the asynchronous phenomenon. It is noted that the Lyapunov function is allowed to increase during some unmatched interval to reduce the conservatism of controller design;
2. Numerically testable asynchronous controller design methods are presented for the dual switching system. The proposed method is suitable for the situation where the asynchronous phenomenon can come from both inaccurate mode detection and time varying delay. Meanwhile, the transition probabilities are both uncertain and partly accessible;
3. Novel asynchronous controller design methods are presented for the semi-Markov jump systems. The sojourn time of the semi-Markov jump systems can have both lower and upper bounds, which could be more practical than previous scenarios.

The organization is as follows: Section 2 formulates the problem. Section 3 proposes the stability and stabilization conditions for the considered two stochastic switching systems. Examples are presented in Section 4. Section 5 concludes the paper. All the proofs are put in the Appendix A.

## 2. Problem Formulation

### 2.1. Problem Formulation for Dual Switching Systems

**Definition** **1.** *Given the following dual switching systems.*

(1) $$\begin{array}{cc} x(k+1) & =A_{g(k),r(k)}x\left(k\right)+B_{g(k),r(k)}u\left(k\right) \end{array}$$

*where $x\left(k\right)\in R^{n}$ is the system state, $u\left(k\right)\in R^{m}$ is the control input.*

*$g\left(k\right)\in M_{1}=\left\{1,2,\ldots ,M_{1}\right\}$ is a deterministic switching law. It is admissible with a average dwell time $\tau_{d}$ [34]. Namely, it satisfies the following condition*

(2) $$N_{g(k)}\left(k_{1},k_{2}\right)\leq N_{0}+\left(k_{2}-k_{1}\right)/\tau_{d}$$

*where $N_{0}\in N$, $\tau_{d}>0$. $N_{g(k)}\left(k_{1},k_{2}\right)$ denotes the switching numbers of g(k) over the time interval $\left[k_{1},k_{2}\right)$.*

*$r\left(k\right)\in M_{2}=\left\{1,2,\ldots ,M_{2}\right\}$ is a homogeneous Markov process defined in the probability space (Ω,F,Pr) where *Ω *is the sample space, F is a σ-field, and Pr is the probability measure. The evolution of r(k) is determined by the transition probability matrix defined as $\Pi_{\nu }≜\left[\pi_{\nu ij}\right],\forall i,j\in M_{2}$ with*

(3) $$\pi_{\nu ij}≜\mathrm{Pr}\left\{r\left(k+1\right)=j|r\left(k\right)=i,g\left(k\right)=\nu \right\}$$

*with $\nu \in M_{1},i,j\in M_{2}$. In practice, the transition probability could suffer from uncertainties and may not be fully accessible. Suppose that*

$${\underline{\pi }}_{\nu ij}\leq \pi_{\nu ij}\leq {\bar{\pi }}_{\nu ij}.$$

*Then, define the following set $M_{2\nu i}=M_{\nu i}^{K}+M_{\nu i}^{UK};M_{\nu i}^{K}\neq ⌀,i\in M_{2}$:*

$$\begin{array}{cc} M_{\nu i}^{K} & =\{j|{\underline{\pi }}_{\nu ij},{\bar{\pi }}_{\nu ij}\;\mathrm{are}\;\mathrm{known}\},\\ M_{\nu i}^{UK} & =\{j|{\underline{\pi }}_{\nu ij},{\bar{\pi }}_{\nu ij},\pi_{\nu ij}\;\mathrm{are}\;\mathrm{unknown}\}. \end{array}$$

*Finally, assume that for each g(k)=ν and r(k)=i, $A_{\nu i}\in R^{n\times n}$ and $B_{\nu i}\in R^{m\times n}$ are known constant matrices.*

In an ideal case, a mode-dependent state feedback controller can be considered for the above system, i.e.,

$$u_{ideal}\left(k\right)=K_{g(k),r(k)}x\left(k\right).$$

However, due to the asynchronous phenomenon, the mode of the dual switching system may not be detected exactly. In this case, we suppose that the actual control effort is expressed as:

(4) $$u\left(k\right)=K_{g\left(k-d\left(k\right)\right),\phi \left(k-\tau_{as}\right)}x\left(k\right)$$

where d(k)∈N is an unknown time varying delay, such that $0\leq d\left(k\right)\leq \tau_{as}\leq \tau_{d}$ with known upper bound $\tau_{as}\in N$. $\phi \left(k\right)\in L=\left\{1,2,\ldots ,L\right\}\subseteq M_{2}$, such that

(5) $$\mu_{\nu i\varphi }=\mathrm{Pr}\left\{\phi \left(k\right)=\varphi |r\left(k\right)=i,g\left(k\right)=\nu \right\}.$$

with $\nu \in M_{1},\varphi \in L,i\in M_{2}$.

**Figure 1:**
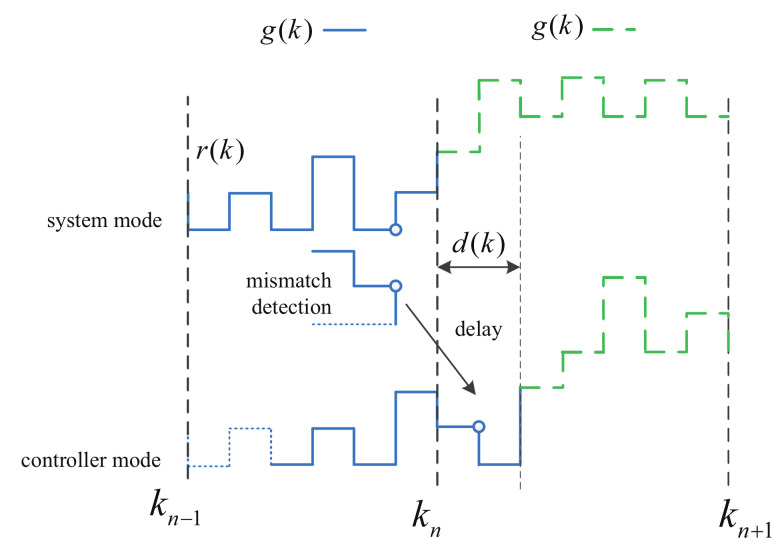
Asynchronous control in dual switching systems.

**Remark** **1.** *As shown in Figure 1, (4) can be interpreted as follows: first when the mode detector detects the mode of the dual switching system, due to the missing measurement, the detected mode may not be the exact current mode. Therefore, a stochastic variable ϕ(k) depending on (5) is presented to describe this pheromone. Second, when the detector transmits the mode information to the controller side, there exists a transmission delay $\tau_{as}$. Note that we assume the deterministic switching mode can be detected exactly. This may lie on that the deterministic g(k) has a larger dwell time $\tau_{d}$ than the r(k). In fact, r(k) may change at every time instance, which implies that it switches much more frequently than g(k). Hence, r(k) is more difficult to be detected, and there may be some mismatch detection. Another reason is that since g(k) is deterministic. One can embed the switching instances of g(k) to the detector in advance. This can improve the accuracy of detection. Additionally, note that the proposed method can be extended to the case where g(k) is not detected exactly.*

**Remark** **2.** *Note that we have assumed that the stochastic switching r(k) represents the fast time-varying conditions, while the deterministic switching g(k) represents the slowly time-varying conditions. For example, consider the servo motor system, the motor may work in different situations, such as no load, external load, external inertia, etc. This can be represented by the deterministic switching. The fast time-varying parameters, which are from stochastic disturbance, abrupt failure, and noise, can be expressed as a stochastic switching sequence. Note that the proposed method can be easily extended to the case when g(k) switches more frequently than r(k). This can be performed by dividing the slowly switching modes into more modes for r(k) intentionally.*

Based on the above analysis, we present our first problem.

**Problem** **1.** *Propose a design method for the asynchronous controller (4), such that the dual switching system (1) is mean square stable.*

### 2.2. Problem Formulation for Semi-Markov Jump Systems

**Definition** **2.** *Given the following semi-Markov jump systems*

(6) $$\begin{array}{cc} x(k+1) & =A_{r(k)}x\left(k\right)+B_{r(k)}u\left(k\right) \end{array}$$

*where $x\left(k\right)\in R^{n}$ and $u\left(k\right)\in R^{m}$ are the same as Definition 1. r(k)∈M={1,2,…,M} is a semi-Markov process and the evolution of it is determined by a semi-Markov kernel (SMK), i.e., $[\Theta_{ij}\left(\tau \right)],\forall i,j\in M$ with*

$$\begin{array}{cc} \Theta_{ij}\left(\tau \right) & =\mathrm{Pr}\{R_{n+1}=j,S_{n}=\tau |R_{n+1}=i\}\\ \; & =\pi_{ij}h_{ij}\left(\tau \right) \end{array}$$

*where i,j∈M, $R_{n}$ represents the mode of system at n-th jump, $S_{n}$ is the sojourn time between (n−1)th jump and nth jump. It is assumed that for the ith mode, its sojourn time $S_{n}^{i}$ has a lower and upper bound like [11], i.e., ${\underline{\tau }}_{i}\leq S_{n}^{i}\leq {\bar{\tau }}_{i},\forall n\in N$ with ${\underline{\tau }}_{i},{\bar{\tau }}_{i}$ being known constants. $\pi_{ij}≜\mathrm{Pr}\left\{r\left(k+1\right)=j|r\left(k\right)=i\right\}$, $h_{ij}\left(\tau \right)≜\mathrm{Pr}\left\{S_{n}=\tau |R_{n}=i,R_{n+1}=j\right\}$ is the sojourn-time probability density function (PDF). Meanwhile, for mode i define function $H_{i}\left(\tau \right)=\mathrm{Pr}\left\{S_{n}\leq \tau |R_{n}=i\right\}$.*

Similar to Section 2.1, the asynchronous controller for the above system is given by

(7) $$u\left(k\right)=K_{r(k-d(k))}x\left(k\right)$$

where d(k)∈N is an uncertain time varying delay, such that $0\leq d\left(k\right)\leq \tau_{as}\leq {\bar{\tau }}_{i},\;i\in M$ with known upper bound $\tau_{as}\in N$.

**Figure 2:**
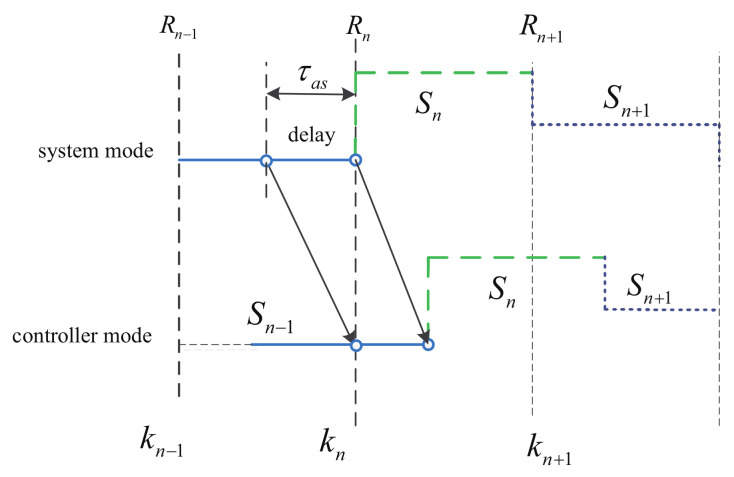
Asynchronous control in semi-Markov jump systems.

**Remark** **3.** *Note that here we only consider the asynchronous phenomena caused by transmission delay. Meanwhile, we consider a small delay effect for the mode detection and a slowly switched law for the semi-Markov jump systems. Hence, compared with the time delay, the sojourn time of the Markov jump systems may be much larger. Please see Figure 2.*

**Definition** **3.** *For the closed loop systems by Definition 2, if the state trajectories satisfy*

$$\lim_{k\to \infty }{E\{||x\left(k\right)||}^{2}{\}|}_{x\left(0\right),r\left(0\right)(S_{n}\in \left[{\underline{\tau }}_{i},{\bar{\tau }}_{i}\right]{|}_{R_{n}=i})}=0.$$

*Then, the system is σ-error mean square stable where σ is defined as $\sigma =\sum_{i\in M}\sigma_{i}$ with $\sigma_{i}≜\left|\ln\left(H_{i}\left({\bar{\tau }}_{i}\right)-H\left({\bar{\tau }}_{i}-1\right)\right)\right|$ denoting the approximation error of the ith mode.*

According to the above analysis, we present our second problem.

**Problem** **2.** *Such that the semi-Markov jump systems (6) are σ-error mean square stable.*

## 3. Main Results

### 3.1. Asynchronous Controller Design for Dual Switching Systems

Substituting (4) into (1), we obtain the closed loop system:

(8) $$\begin{array}{cc} x(k+1) & =\left(A_{g(k),r(k)}+B_{g(k),r(k)}K_{g\left(k-\tau_{as}\right),\phi \left(k-\tau_{as}\right)}\right)x\left(k\right)\\ \; & =\left(A_{\nu i}+B_{\nu i}K_{\hat{\nu }\hat{\varphi }}\right)x\left(k\right)\\ \; & =A_{\nu \hat{\nu }j\hat{\varphi }}x\left(k\right) \end{array}$$

where $\hat{\nu },\nu \in M_{1}$, $j,\hat{\varphi }\in M_{2}$, $A_{\nu \hat{\nu }j\hat{\varphi }}=A_{\nu i}+B_{\nu i}K_{\hat{\nu }\hat{\varphi }}.$

To handle the asynchronous phenomenon in (8), we first present the following stability criterion.

**Lemma** **1.** *For the system (8), suppose there exists $C^{1}$ Lyapunov functions $V_{g(k),r(k)}\left(x\left(k\right)\right):R^{n}\to R,g\left(k\right)\in M_{1},r\left(k\right)\in M_{2}$ such that for any $\hat{\nu },\nu \in M_{1}$, $j,j_{1},j_{2},\ldots ,j_{\tau_{as}-1},i\in M_{2}$,*

$$K_{1}\left(||x\left(k\right)||\right)\leq V_{\nu i}\left(x\left(k\right)\right)\leq K_{2}\left(||x\left(k\right)||\right),$$

(9) $$\begin{array}{cc} \; & E\left[V_{g(k+1),r(k+1)}\left(x\left(k+1\right)\right)\right]{|}_{x\left(k\right),\bar{r}(k)=\left[j,j_{1},\ldots ,j_{d(k)-1},i\right]}\\ \leq \; & E\left[\chi \left(k\right)V_{g(k),r(k)}\left(x\left(k\right)\right)\right]{|}_{x\left(k\right),\bar{r}(k)=\left[j,j_{1},\ldots ,j_{d(k)-1},i\right]},\\ \; & \forall k\in N[k_{n},k_{n+1}), \end{array}$$

(10) $$V_{\hat{\nu }i}\left(x\left(k_{n}\right)\right)\leq \lambda V_{\nu i}\left(x\left(k_{n}\right)\right)$$

*where λ>1; $K_{1}\left(\cdot \right)$ and $K_{2}\left(\cdot \right)$ are two $K_{\infty }\left(\cdot \right)$ functions; $k_{n}$ with n∈N denotes switching instances for the signal g(k). $\bar{r}\left(k\right)$ is a vector of the previous system modes and given by*

$$\bar{r}(k)=\left[r\left(k\right),r\left(k-1\right),\ldots ,r\left(k-d\left(k\right)\right)\right].$$

*For $k\in N\cap [k_{n},k_{n+1})$ χ(k)∈R satisfying*

(11) $$\chi \left(k\right)=\left\{\begin{array}{cc} \alpha , & if\;\;r(k)\neq r(k-d(k)),\\ \beta , & if\;\;r(k)=r(k-d(k)), \end{array}\right.$$

*with α>1 and 0<β<1 being two positive constants.*

*Then, the system (8) is mean square stable for any switching signal g(k) with average dwell time*

(12) $$\tau_{d}>\tau_{d}^{*}=-\left[\tau_{as}\left(\ln\alpha -\ln\beta \right)+\ln\lambda \right]/\ln\beta .$$

Based on the above lemma, we have the following theorem in terms of matrix inequalities.

**Lemma** **2.** *The following statements (i)–(iii) satisfy*

(iii)⇔(ii)⇒(i).

*(i) The system (8) is mean square stable with dwell switching signal g(k) satisfying (12).*

*(ii) There exist matrices $T_{\nu \hat{\nu }ij\hat{\varphi }}≻0$, $P_{\sigma j}≻0$ such that for any $\hat{\nu },\nu \in M_{1}$, $i,j,\hat{\varphi }\in M_{2}$,*

(13) $$\begin{array}{cc} \; & \sum_{\hat{\varphi }\in L}\mu_{\hat{\nu }i\hat{\varphi }}A_{\nu \hat{\nu }j\hat{\varphi }}^{T}P_{\nu j}^{K}A_{\nu \hat{\nu }j\hat{\varphi }}\\ + & \sum_{\hat{\varphi }\in L}\mu_{\hat{\nu }i\hat{\varphi }}A_{\nu \hat{\nu }j\hat{\varphi }}^{T}P_{\nu j}^{UK}A_{\nu \hat{\nu }j\hat{\varphi }}-{\bar{\chi }}_{\nu \hat{\nu }}P_{\nu j}≺0, \end{array}$$

$$P_{\nu j}^{K}=\sum_{l\in M_{\nu i}^{K}}{\bar{\pi }}_{\nu jl}P_{\nu l},$$

$$P_{\nu j}^{UK}=\left(1-\sum_{l\in M_{\nu i}^{K}}{\underline{\pi }}_{\nu jl}\right)\sum_{l\in M_{\nu i}^{UK}}P_{\nu l},$$

(14) $$P_{\hat{\nu }i}⪯\lambda P_{\nu i}.$$

*where λ>1 and ${\bar{\chi }}_{\nu \hat{\nu }}$ is calculated as:*

(15) $${\bar{\chi }}_{\nu \hat{\nu }}=\left\{\begin{array}{cc} \alpha , & if\;\;\nu \neq \hat{\nu },\\ \beta , & if\;\;\nu =\hat{\nu }, \end{array}\right.$$

*with α>1 and 0<β<1 being two positive constants.*

*(iii) There exist matrices $T_{\nu \hat{\nu }ij\hat{\varphi }}≻0$, $P_{\sigma j}≻0$ such that for any $\hat{\nu },\nu \in M_{1}$, $i,j,\hat{\varphi }\in M_{2}$,*

(16) $$\left[\begin{array}{cc} -T_{\nu \hat{\nu }ij\hat{\varphi }} & A_{\nu \hat{\nu }j\hat{\varphi }}\\ ⋆ & -D_{\nu } \end{array}\right]≺0,$$

(17) $$\sum_{\hat{\varphi }=1}^{M_{2}}\mu_{\hat{\nu }i\hat{\varphi }}T_{\nu \hat{\nu }ij\hat{\varphi }}-{\bar{\chi }}_{\nu \hat{\nu }}P_{\nu j}≺0,$$

$$P_{\hat{\nu }i}⪯\lambda P_{\nu j}$$

*where $D_{\nu }=\mathrm{diag}\left\{P_{\nu 1}\;P_{\nu 2}\;\ldots \;P_{\nu M_{2}}\right\}$, $A_{\nu \hat{\nu }j\hat{\varphi }}=\left[\sqrt{{\tilde{\pi }}_{\nu j1}}A_{\nu \hat{\nu }j\hat{\varphi }}^{T}P_{\nu 1}\;\sqrt{{\tilde{\pi }}_{\nu j2}}A_{\nu \hat{\nu }j\hat{\varphi }}^{T}P_{\nu 2}\;\ldots \;\sqrt{{\tilde{\pi }}_{\nu jL}}A_{\nu \hat{\nu }j\hat{\varphi }}^{T}P_{\nu M_{2}}\right]$. λ and ${\bar{\chi }}_{\nu \hat{\nu }}$ are the same as statement (ii).*

*${\tilde{\pi }}_{\nu jl},l\in M_{2}$ is defined as:*

(18) $${\tilde{\pi }}_{\nu jl}=\left\{\begin{array}{cc} {\bar{\pi }}_{\nu jl}, & if\;\;l\in M_{\nu i}^{K},\\ 1-\sum_{l\in M_{\nu i}^{K}}{\underline{\pi }}_{\nu jl}, & if\;\;l\in M_{\nu i}^{UK}. \end{array}\right.$$

**Remark** **4.** *It is noted that statements (ii) and (iii) are equivalent and can be both used to check the stability of dual switching systems with asynchronous phenomenon and uncertain probability transition rates. However, statements (iii) are in strict LMI and can be solved efficiently.*

Based on the above result, we can compute the control gain in (4).

**Theorem** **1.** *For the system (8), if there exist matrices ${\bar{T}}_{\nu \hat{\nu }ij\hat{\varphi }}≻0$, ${\bar{P}}_{\nu j}≻0$, $Q_{\nu i}≻0$, $K_{\hat{\nu }\hat{\varphi }}$, $G_{\hat{\nu }\hat{\varphi }}$, such that for any $\hat{\nu },\nu \in M_{1}$, $i,j,\hat{\varphi }\in M_{2}$, $\kappa \in [1,\tau_{as}-1]$,*

(19) $$\left[\begin{array}{cc} {\bar{T}}_{\nu \hat{\nu }ij\hat{\varphi }}-G_{\hat{\nu }\hat{\varphi }}-G_{\hat{\nu }\hat{\varphi }}^{T} & B_{\nu \hat{\nu }j\hat{\varphi }}\\ ⋆ & -{\bar{D}}_{\nu } \end{array}\right]≺0,$$

(20) $$\left[\begin{array}{cc} {\bar{\chi }}_{\nu \hat{\nu }}{\bar{P}}_{\nu j}-2{\bar{\chi }}_{\nu \hat{\nu }}Q_{\nu \hat{\nu }ij\hat{\varphi }} & Q_{\nu \hat{\nu }ij\hat{\varphi }}\\ ⋆ & -T_{\nu i} \end{array}\right]≺0,$$

$${\bar{P}}_{\nu i}⪯\lambda {\bar{P}}_{\hat{\nu }i}$$

*where λ, ${\bar{\chi }}_{\nu \hat{\nu }}$ and ${\tilde{\pi }}_{\nu jl},l\in M_{2}$, $\hat{\varphi }\in L$ are the same as Theorem 2. ${\bar{D}}_{\nu }=\mathrm{diag}\left\{{\bar{P}}_{\nu 1}\;{\bar{P}}_{\nu 2}\;\ldots \;{\bar{P}}_{\nu M_{2}}\right\}$, $T_{\nu i}=\mathrm{diag}\left\{{\bar{T}}_{\nu \hat{\nu }ij1}\;{\bar{T}}_{\nu \hat{\nu }ij2}\;\ldots \;{\bar{T}}_{\nu \hat{\nu }ijM_{2}}\right\}$,*

$$B_{\nu \hat{\nu }j\hat{\varphi }}=\left[\sqrt{{\tilde{\pi }}_{\nu j1}}\left({\left(A_{\nu r}G_{\hat{\nu }\hat{\varphi }}\right)}^{T}+{\left(B_{\nu r}K_{\hat{\nu }\hat{\varphi }}\right)}^{T}\right)\;\ldots \;\sqrt{{\tilde{\pi }}_{\nu jM_{2}}}\left({\left(A_{\nu r}G_{\hat{\nu }\hat{\varphi }}\right)}^{T}+{\left(B_{\nu r}K_{\hat{\nu }\hat{\varphi }}\right)}^{T}\right)\right],$$

$$Q_{\nu \hat{\nu }ij\hat{\varphi }}=\left[\sqrt{\mu_{\hat{\nu }i1}}Q_{\nu \hat{\nu }ij\hat{\varphi }}\;\sqrt{\mu_{\hat{\nu }i2}}Q_{\nu \hat{\nu }ij\hat{\varphi }}\;\ldots \;\sqrt{\mu_{\hat{\nu }iM_{2}}}Q_{\nu \hat{\nu }ij\hat{\varphi }}\right].$$

*Then, there exists a set of stabilizing controllers, such that (1) is mean square stable for dwell switching signal g(k) satisfying (12). The admissible controller can be given by:*

$$K_{\hat{\nu }\hat{\varphi }}=K_{\hat{\nu }\hat{\varphi }}G_{\hat{\nu }\hat{\varphi }}^{-1}.$$

**Remark** **5.** *Compared with [25,37,38] the proposed stability criterion and controller design method can be used to simultaneously handle dual switching dynamics (switching sequence g(k),r(k)), mismatch mode detection (ϕ(k)), and mode transmission delay ($\tau_{as}$). This is achieved by using a multiple Lyapunov function technique (see the proof of Lemma 1). It is noted that the conditional expectation of the Lyapunov function is allowed to increase from (9). This is more general than the existing studies [25,37,38]. Meanwhile, different from [25,37,38], the condition for the expectation of the Lyapunov function is not only dependent on the latest mode r(k−1) but also on the latest $\tau_{as}$ mode $r\left(k-1\right),\ldots ,r\left(k-\tau_{as}\right)$. This will bring more difficulties to the controller design.*

### 3.2. Asynchronous Controller Design for Semi-Markov Systems

Substituting (7) into (6), we obtain the closed loop system:

(21) $$\begin{array}{cc} x(k+1) & =\left(A_{r(k)}+B_{r(k)}K_{r(k-\tau_{as})}\right)x\left(k\right)\\ \; & =\left(A_{j}+B_{j}K_{i}\right)x\left(k\right)\\ \; & =A_{ij}x\left(k\right) \end{array}$$

where i,j∈M.

To handle the asynchronous phenomenon in (21), we first present the following stability criterion.

**Lemma** **3.** *For the system (21), suppose there exists mode-dependent Lyapunov functions $V_{r(k)}\left(x\left(k\right),k-k_{n}\right):R^{n}\to R,r\left(k\right)\in M,k\in N\cap \left[k_{n},k_{n+1}-1\right]$, such that for ∀i,j∈M,*

$$K_{1}\left(||x\left(k\right)||\right)\leq V_{r(k_{n})}\left(x\left(k\right),k-k_{n}\right)\leq K_{2}\left(||x\left(k\right)||\right),$$

(22) $$\begin{array}{cc} \; & E\left[V_{r(k_{n})}\left(x\left(k\right),k-k_{n}\right)\right]{|}_{x\left(k\right),r\left(k_{n}\right)=j,r\left(k_{n-1}\right)=i}\\ \leq \; & E\left[\rho_{r(k_{n})}V_{r(k_{n})}\left(x\left(k_{n}\right),0\right)\right]|{}_{x\left(k\right),r\left(k_{n}\right)=j,r\left(k_{n-1}\right)=i}\\ \; & k\in N[k_{n}+1,k_{n+1}-1], \end{array}$$

(23) $$\begin{array}{cc} \; & E\left[V_{r(k_{n+1})}\left(x\left(k_{n+1}\right),0\right)\right]{|}_{x\left(k\right),r\left(k_{n}\right)=j,r\left(k_{n-1}\right)=i}\\ -\; & E\left[V_{r(k_{n})}\left(x\left(k_{n}\right),0\right)\right]{|}_{x\left(k\right),r\left(k_{n}\right)=j,r\left(k_{n-1}\right)=i}\leq K_{3}(||x\left(k\right)\left||\right) \end{array}$$

*where $K_{1}\left(\cdot \right)$, $K_{2}\left(\cdot \right)$, and $K_{3}\left(\cdot \right)$ are all $K_{\infty }\left(\cdot \right)$ functions, $\rho_{r(k_{n})}$ are finite positive constants.*

*Then, the system (21) is σ-error mean square stable.*

**Remark** **6.** *Compared with [11,28], the condition for the expectation of the Lyapunov function is not only dependent on the latest mode $r(k_{n})$ but also on the $r(k_{n-1})$. This will bring difficulties to the controller design.*

Based on the above lemma, we will present the stability criterion in terms of matrix inequalities.

**Lemma** **4.** *Given matrices $P_{j}\left(\zeta \right)≻0$, j∈M, $\zeta \in N\cap [0,{\bar{\tau }}_{j}-1]$, the closed loop system (21) is σ-error mean square stable if*

(24) $$\left(A_{i_{1}j}A_{i_{2}j}\cdots A_{i_{\zeta }j}\right){}^{T}P_{j}\left(\zeta \right)A_{i_{1}j}A_{i_{2}j}\cdots A_{i_{\zeta }j}-\rho_{j}P_{j}\left(0\right)≺0,$$

(25) $$\sum_{\tau ={\underline{\tau }}_{j}}^{{\bar{\tau }}_{j}}\left(A_{i_{1}j}\cdots A_{i_{\tau }j}\right){}^{T}P_{j}\left(\tau \right)A_{i_{1}j}\cdots A_{i_{\tau }j}-P_{j}\left(0\right)≺0,$$

*for any i,j∈M, $\forall \zeta \in N\cap [1,{\bar{\tau }}_{j}-1],$ $\forall i_{\zeta }≜\left\{i_{1},i_{2},\ldots ,i_{\zeta }\right\}\in I_{\zeta }^{ij}$, $\forall i_{\tau }≜\left\{i_{1},i_{2},\ldots ,i_{\tau }\right\}\in I_{\tau }^{ij}$ where $P_{j}\left(\tau \right)=\sum_{l=1}^{M}\Theta_{jl}\left(\tau \right)P_{l}\left(0\right)$, $I_{\chi }^{ij}$ with χ=ζ or τ are defined as follows.*

*If $\tau_{as}\leq {\underline{\tau }}_{i},\;i\in M$,*

(26) $$\begin{array}{cc} I_{\chi }^{ij}= & \{i_{\zeta }|i_{1}=i_{2}=\ldots =i_{\chi -\tau_{as}}=j;\\ \; & \;\;i_{\chi -\tau_{as}+1},i_{\chi -\tau_{as}+2},\ldots ,i_{\chi }\in \left\{i,j\right\}\}\;for\;\;\chi \geq \tau_{as}; \end{array}$$

(27) $$I_{\chi }^{ij}=\left\{i_{\chi }|i_{1},i_{2},\ldots ,i_{\chi }\in \left\{i,j\right\}\right\}\;for\;\;1\leq \chi <\tau_{as}.$$

*If $\tau_{as}>{\underline{\tau }}_{i},\;i\in M$,*

(28) $$\begin{array}{cc} I_{\chi }^{ij}= & \{i_{\zeta }|i_{1}=i_{2}=\ldots =i_{\chi -\tau_{as}}=j;\\ \; & \;\;i_{\chi -\tau_{as}+1},i_{\chi -\tau_{as}+2},\ldots ,i_{\chi -\tau_{as}+{\underline{\tau }}_{i}}\in \left\{i,j\right\};\\ \; & \;\;i_{\chi -\tau_{as}+{\underline{\tau }}_{i}+1},\ldots ,i_{\chi }\in M\}\;for\;\;\chi \geq \tau_{as}; \end{array}$$

(29) $$\begin{array}{cc} I_{\chi }^{ij}= & \{i_{\chi }|i_{1},i_{2},\ldots ,i_{{\underline{\tau }}_{i}}\in \left\{i,j\right\};\\ \; & \;\;i_{{\underline{\tau }}_{i}+1},\ldots ,i_{\chi }\in M\}\;for\;\;\tau_{as}-{\underline{\tau }}_{i}+1\leq \chi <\tau_{as}; \end{array}$$

(30) $$I_{\chi }^{ij}=\left\{i_{\chi }|i_{1},i_{2},\ldots ,i_{\chi }\in M\right\}\;for\;\;1\leq \chi \leq \tau_{as}-{\underline{\tau }}_{i}.$$

*Specifically, if $d\left(k\right)=\tau_{as}$, then*

*If $\tau_{as}\leq {\underline{\tau }}_{i},\;i\in M$,*

(31) $$\begin{array}{cc} I_{\chi }^{ij}= & \{i_{\zeta }|i_{1}=i_{2}=\ldots =i_{\chi -\tau_{as}}=j;\\ \; & \;\;i_{\chi -\tau_{as}+1}=i_{\chi -\tau_{as}+2}=\ldots =i_{\chi }=i\}\;for\;\;\chi \geq \tau_{as}; \end{array}$$

(32) $$I_{\chi }^{ij}=\left\{i_{\chi }|i_{1},i_{2},\ldots ,i_{\chi }\in \left\{i,j\right\}\right\}\;for\;\;1\leq \chi <\tau_{as}.$$

*If $\tau_{as}>{\underline{\tau }}_{i},\;i\in M$,*

(33) $$\begin{array}{cc} I_{\chi }^{ij}= & \{i_{\zeta }|i_{1}=i_{2}=\ldots =i_{\chi -\tau_{as}}=j;\\ \; & \;\;i_{\chi -\tau_{as}+1}=i_{\chi -\tau_{as}+2}=\ldots =i_{\chi -\tau_{as}+{\underline{\tau }}_{i}}=i;\\ \; & \;\;i_{\chi -\tau_{as}+{\underline{\tau }}_{i}+1},\ldots ,i_{\chi }\in M\}\;for\;\;\chi \geq \tau_{as}; \end{array}$$

(34) $$\begin{array}{cc} I_{\chi }^{ij}= & \{i_{\chi }|i_{1}=i_{2}=\ldots =i_{{\underline{\tau }}_{i}}=i;\\ \; & \;\;i_{{\underline{\tau }}_{i}+1},\ldots ,i_{\chi }\in M\}\;for\;\;\tau_{as}-{\underline{\tau }}_{i}+1\leq \chi <\tau_{as}; \end{array}$$

(35) $$I_{\chi }^{ij}=\left\{i_{\chi }|i_{1},i_{2},\ldots ,i_{\chi }\in M\right\}\;for\;\;1\leq \chi \leq \tau_{as}-{\underline{\tau }}_{i}.$$

**Remark** **7.** *According to whether $\tau_{as}\leq {\underline{\tau }}_{i}$ or not, $I_{\chi }^{ij}$ is defined separately. Meanwhile, as stated in Remark 3, we mainly consider a small delay effect and slowly switched law for the semi-Markov jump systems. Therefore, there is a high probability that $\tau_{as}\leq {\bar{\tau }}_{i},\;i\in M$. Thus, (26)–(27) and (31)–(32) are applicable.*

The above result can be converted into strict LMI form.

**Lemma** **5.** *Given matrices $\Phi_{j}\left(\zeta ,p\right)≻0$, $\Psi_{j}\left(\tau ,q\right)≻0$, $T_{ij\tau }≻0$ where i,j∈M; ζ,p,τ,q∈N are integers lying in intervals $\left[0,{\bar{\tau }}_{j}-1\right]$, [0,ζ], $\left[{\underline{\tau }}_{j},{\bar{\tau }}_{j}\right]$ and [0,τ], respectively. Then, the closed loop system (21) is σ-error mean square stable if*

(36) $$\left[\begin{array}{cc} -\Phi_{j}\left(\zeta ,p\right) & A_{i_{p+1}j}^{T}\Phi_{j}\left(\zeta ,p+1\right)\\ ⋆ & -\Phi_{j}\left(\zeta ,p+1\right) \end{array}\right]≺0,$$

(37) $$\Phi_{j}\left(\zeta ,0\right)-\rho_{j}\Phi_{j}\left(0,0\right)≺0,$$

(38) $$\left[\begin{array}{cc} -\Psi_{j}\left(\tau ,\tau -1\right) & A(\tau )\\ ⋆ & -\Phi \end{array}\right]≺0,$$

(39) $$\left[\begin{array}{cc} -\Psi_{j}\left(\tau ,q\right) & A_{i_{q+1}j}^{T}\Psi_{j}\left(\tau ,q+1\right)\\ ⋆ & -\Psi_{j}\left(\tau ,q+1\right) \end{array}\right]≺0,$$

(40) $$\Psi_{j}\left(\tau ,0\right)-T_{ij\tau }≺0,$$

(41) $$\sum_{\tau ={\underline{\tau }}_{j}}^{{\bar{\tau }}_{j}}T_{ij\tau }-\Phi_{j}\left(0,0\right)≺0$$

*for any i,j∈M, ∀ζ,p,τ,q in intervals $\left[0,{\bar{\tau }}_{j}-1\right]$, [0,ζ−1], $\left[{\underline{\tau }}_{j},{\bar{\tau }}_{j}\right]$ and [0,τ−2], respectively, $\forall i_{\zeta }≜\left\{i_{1},i_{2},\ldots ,i_{\zeta }\right\}\in I_{\zeta }^{j}$, $\forall i_{\tau }≜\left\{i_{1},i_{2},\ldots ,i_{\tau }\right\}\in I_{\tau }^{j}$ where $I_{\chi }^{ij}$ with χ=ζ or τ are defined as Lemma 4,*

$$\Phi =\mathrm{diag}\left\{\begin{array}{cccc} \Phi_{1}\left(0,0\right) & \Phi_{2}\left(0,0\right) & \ldots & \Phi_{M}\left(0,0\right) \end{array}\right\},$$

$$A\left(\tau \right)=[\sqrt{\Theta_{j1}\left(\tau \right)}A_{jj}^{T}\Psi_{1}\left(0,0\right)\;\ldots \;\sqrt{\Theta_{jM}\left(\tau \right)}A_{jj}^{T}\Psi_{M}\left(0,0\right)].$$

Based on the above lemma, we can compute the control gain in (7).

**Theorem** **2.** *Given matrices ${\tilde{\Phi }}_{j}\left(\zeta ,p\right)≻0$, ${\tilde{\Psi }}_{j}\left(\zeta ,q\right)≻0$, ${\tilde{T}}_{ij\tau }≻0$, $R_{i\zeta }≻0$, $K_{j}$, $G_{j}$ where i,j∈M; ζ,p,τ,q∈N are integers lying in intervals $\left[0,{\bar{\tau }}_{j}-1\right]$, [0,ζ], $\left[{\underline{\tau }}_{j},{\bar{\tau }}_{j}\right]$ and [0,τ], respectively. Suppose*

(42) $$\left[\begin{array}{cc} {\tilde{\Phi }}_{j}\left(\zeta ,p\right)-G_{i_{p+1}} & {\left(A_{j}G_{i_{p+1}}\right)}^{T}+{\left(B_{j}K_{i_{p+1}}\right)}^{T}\\ ⋆ & -{\tilde{\Phi }}_{j}\left(\kappa ,p+1\right) \end{array}\right]≺0,$$

(43) $${\tilde{\Phi }}_{j}\left(0,0\right)-\rho_{j}{\tilde{\Phi }}_{j}\left(\zeta ,0\right)≺0,$$

(44) $$\left[\begin{array}{cc} {\tilde{\Psi }}_{j}\left(\tau ,\tau -1\right)-G_{j}-G_{j}^{T} & B(\tau )\\ ⋆ & -\tilde{\Phi } \end{array}\right]≺0,$$

(45) $$\left[\begin{array}{cc} {\tilde{\Psi }}_{j}\left(\tau ,q\right)-G_{i_{q+1}} & {\left(A_{j}G_{i_{q+1}}\right)}^{T}+{\left(B_{j}K_{i_{q+1}}\right)}^{T}\\ ⋆ & -{\tilde{\Psi }}_{j}\left(\tau ,q+1\right) \end{array}\right]≺0,$$

(46) $${\tilde{T}}_{ij\tau }-{\tilde{\Psi }}_{j}\left(\tau ,0\right)≺0,$$

(47) $$\left[\begin{array}{cc} {\tilde{\Phi }}_{j}\left(0,0\right)-2Q_{ij\tau } & Q_{ij\tau }\\ ⋆ & -{\tilde{T}}_{i} \end{array}\right]≺0$$

*hold for any i,j∈M, ∀ζ,p,τ,q in intervals $\left[0,{\bar{\tau }}_{j}-1\right]$, [0,ζ−1], $\left[{\underline{\tau }}_{j},{\bar{\tau }}_{j}\right]$ and [0,τ−2], respectively, $\forall i_{\zeta }≜\left\{i_{1},i_{2},\ldots ,i_{\zeta }\right\}\in I_{\zeta }^{j}$, $\forall i_{\tau }≜\left\{i_{1},i_{2},\ldots ,i_{\tau }\right\}\in I_{\tau }^{j}$ where $I_{\chi }^{ij}$ with χ=ζ or τ are defined as Lemma 4,*

$$G_{l}=G_{l}+G_{l}^{T}\left(l\in M\right)$$

$$B\left(\tau \right)=\left[\sqrt{\Theta_{j1}\left(\tau \right)}V_{j}\;\sqrt{\Theta_{j2}\left(\tau \right)}V_{j}\;\ldots \;\sqrt{\Theta_{jM}\left(\tau \right)}V_{j}\right],$$

$$V_{j}={\left(A_{j}G_{j}\right)}^{T}+{\left(B_{j}K_{j}\right)}^{T},$$

$$Q_{ij\tau }=\left[Q_{ij\tau }\;Q_{ij\tau }\;\ldots ,Q_{ij\tau }\right],$$

$${\tilde{T}}_{i}=\mathrm{diag}\left\{\begin{array}{cccc} {\tilde{T}}_{ij{\underline{\tau }}_{j}} & {\tilde{T}}_{ij,{\underline{\tau }}_{j}+1} & \ldots & {\tilde{T}}_{ij{\bar{\tau }}_{j}} \end{array}\right\}.$$

*Then, there exists a set of stabilizing controllers, such that (21) is σ-error mean square stable. The admissible controller can be given by:*

$$K_{j}=K_{j}G_{j}^{-1}.$$

**Remark** **8.** *In contrast with the methods in [11,28,29,30], the proposed method has three distinguishing features: (1) asynchronous stabilizing controllers are designed which fully consider the effect of mode transmission delay. This can be seen from r(k−d(k)) in (7), which is different from the controller in [11,28,29,30]; (2) it is suitable for the semi-Markov jump systems, of which the sojourn time can have both lower and upper bounds (${\underline{\tau }}_{i},{\bar{\tau }}_{i}$ in semi-Markov kernel). Note that, in order to handle both the sojourn time and the time delay, we have introduced the definition $I_{\chi }^{ij}$ in Lemma 4. This is the key for tackle this issue; (3) some auxiliary matrix variables $T_{ij\tau }$ are introduced to transform (24)–(25) into (36)–(41) and (42)–(47). This may result in a simpler controller design criterion in terms of strict LMI.*

**Remark** **9.** *We have only considered the stabilization problem for semi-Markov jump systems. However, the proposed method can be extended to solve the controller design problem under performance constraints, such as mixed $H_{\infty }$ and passivity [3,4]. Nevertheless, note that the state equation of the semi-Markov jump system needs to be iterated from time instant k to time $k+\tau_{i}$ to describe the relationship between Lyapunov functions at time instant k to time $k+\tau_{i}$. In this case, the external disturbance terms in the system may bring some difficulties.*

## 4. Examples

**Example** **1.** *Consider a DC motor system described in [37]. It is expressed by Definition 1. The DC motor is driven by the traditional speed loop controller [6,40]. The state variables $x_{1}$, $x_{2}$ represent the velocity and current of the DC motor, respectively. g(k) has two modes, which represent that the DC motors are working in two conditions with different loads. r(k) has three modes, which corresponds to (1) 0% of rotary (normal mode); (2) +20% of rotary for improving the power (low mode); and (3) −40% of rotary for decreasing the power (medium mode). The initial conditions are selected as x(0)=[1,2,1] and r(0) is randomly generated from the set $M_{2}$. The system matrices in (1) are given by: $A_{11}=$*[*−0.4799 5.15460;−3.816214.47320;0.1399 0 −0.9255], $A_{12}=$ [−1.6026 9.1632 0; −0.5918 3.0317 0; 0.0740 0 −0.4338], $A_{13}=$ [0.6346 0.9178 0; −0.5056 2.4811 0; 0.3865 0 0.0982]; $A_{21}=$*[*−0.7 5 0; −4 14 0; 0.2 0 −1.5], $A_{12}=$ [−1.5 9 0; −0.6 3 0; 0.1 0 −0.4], $A_{13}=$ [0.5 1 0; −0.5 2.5 0; 0.4 0 0.1]; $B_{21}=B_{11}={\left[5.8705\;15.5010\;0\right]}^{T}$, $B_{22}=B_{12}={\left[10.2951\;2.2282\;0\right]}^{T},$ and $B_{23}=B_{13}={\left[0.7874\;1.5302\;0\right]}^{T}$.*

The transition probability matrices are given by:

$$\Pi_{1}=\left[0\;0.4\;0.6;0.5\;0\;0.5;0.7\;0.3\;0\right],\;\Pi_{2}=\left[0\;0.3\;0.7;0.4\;0\;0.6;0.9\;0.1\;0\right].$$

We also assume that these two matrices are not known exactly. The accessible information about these two matrices are as follows:

$$\Pi_{1}=\left[\begin{array}{ccc} 0 & 0.4 & 0.6\\ {}[0.5,0.6] & 0 & ?\\ {}[0.7,0.8] & ? & 0 \end{array}\right],\;\Pi_{2}=\left[\begin{array}{ccc} 0 & \left[0.3,0.4\right] & ?\\ {}[0.4,0.5] & 0 & ?\\ 0.9 & ? & ? \end{array}\right].$$

**Figure 3:**
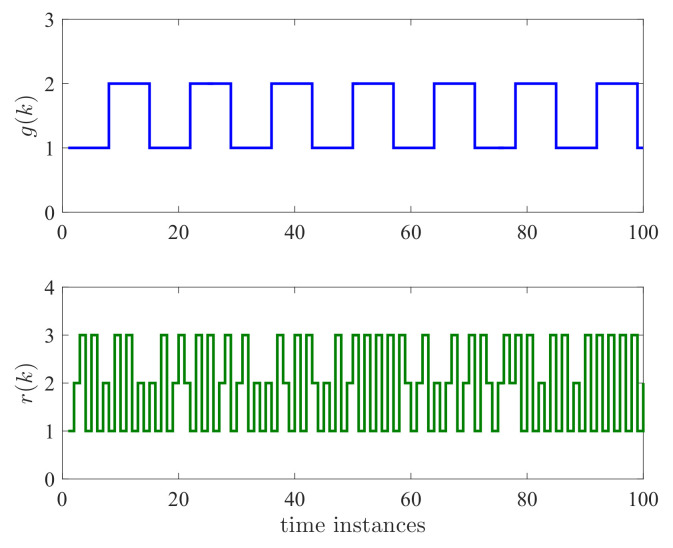
Switching signals g(k) and r(k).

The switching signal g(k) and r(k) are shown in Figure 3. It can be seen that g(k) has an average dwell time $\tau_{d}=7$ and r(k) is a stochastic process. Hence, the considered system contains both deterministic and stochastic dynamics.

In order to stabilize the DC motor system, the asynchronous controller (4) is utilized. Let $\alpha_{1}=\alpha_{2}=\alpha_{3}=1.1$, $\beta_{1}=0.92,\beta_{2}=0.90,\beta_{3}=0.95$, $\lambda_{1}=1.1,\lambda_{2}=1.2,\lambda_{3}=1.1$, $\tau_{as}=2$ in Theorem 1. It can be verified that for this parameters $6.5=\tau_{d}^{*}<\tau_{d}$. Then, we can compute the mode-dependent control gain by solving the LMI in (19) and (20). The solutions of the LMI are given by $K_{11}={\left[-0.1036\;-0.4025\;0.2260\right]}^{T}\times 10^{-10},$ $K_{21}={\left[-0.1040\;-0.3813\;0.2419\right]}^{T}\times 10^{-10},$ $G_{11}=\left[\begin{array}{ccc} 0.2635 & 0.2114 & -0.0976\\ 0.1850 & 0.5070 & -0.2749\\ -0.0570 & -0.2487 & 0.8192 \end{array}\right]\times 10^{-10},$ $G_{21}=\left[\begin{array}{ccc} 0.2612 & 0.1933 & -0.0914\\ 0.1842 & 0.4801 & -0.2892\\ -0.0530 & -0.2347 & 0.8201 \end{array}\right]\times 10^{-10}.$ Other modes are similar. The control gains are given as $K_{11}={\left[0.2313\;-0.8876\;0.0056\right]}^{T},$ $K_{21}={\left[0.2244\;-0.8798\;0.0098\right]}^{T},$ $K_{12}={\left[0.2306\;-0.8873\;0.0064\right]}^{T},$ $K_{22}={\left[0.2244\;-0.8796\;0.0098\right]}^{T},$ $K_{13}={\left[0.2306\;-0.8873\;0.0064\right]}^{T},$ and $K_{23}={\left[0.2229\;-0.8802\;0.0094\right]}^{T}$.

**Figure 4:**
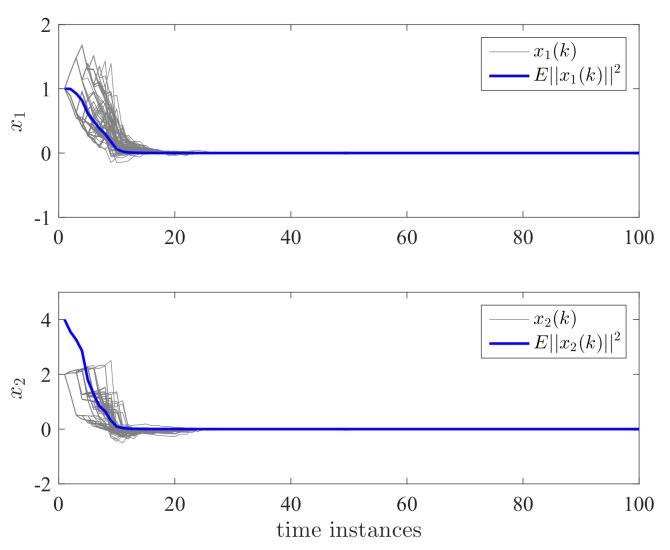
State responses $x_{1}$ and $x_{2}$ for dual switching systems.

**Figure 5:**
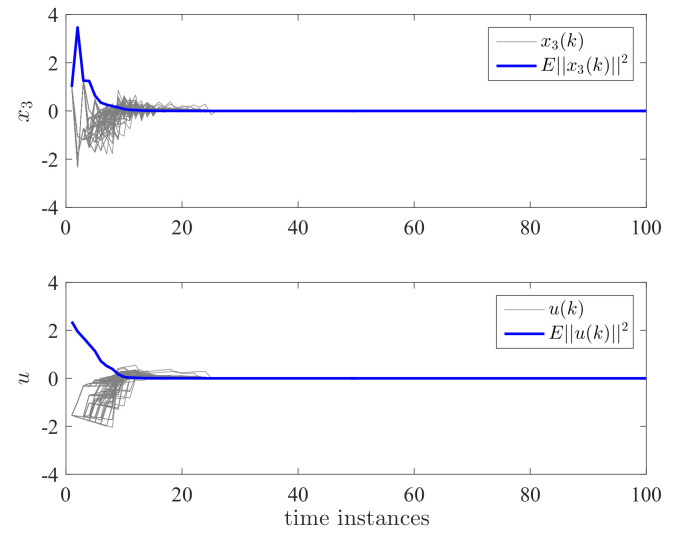
State responses $x_{3}$ and control effort *u* for dual switching systems.

**Figure 6:**
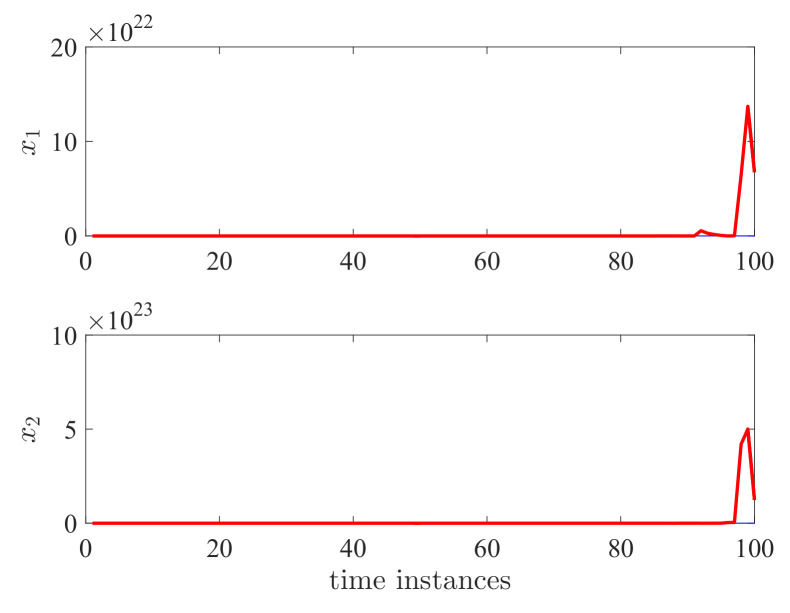
Performance comparison for state responses $x_{1}$ and $x_{2}$.

**Figure 7:**
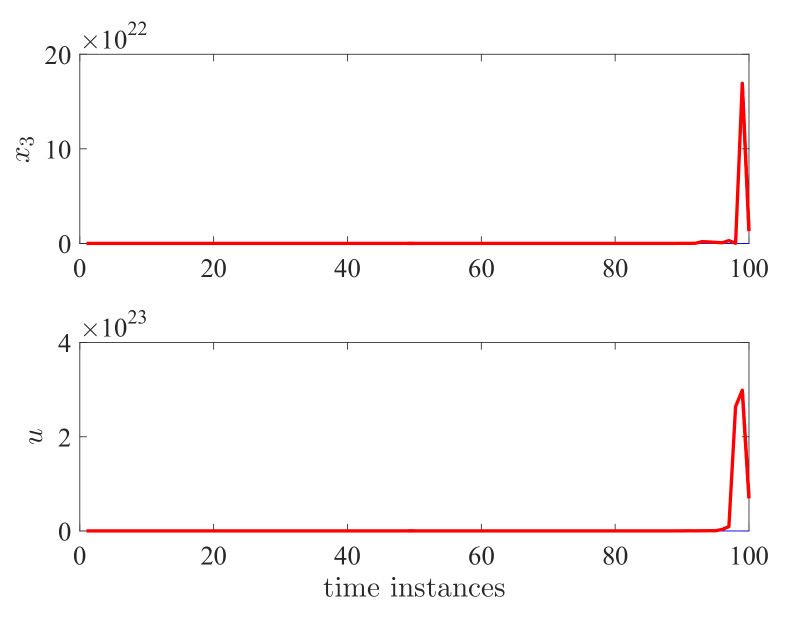
Performance comparison for state responses $x_{3}$ and *u*.

By applying the proposed controller for the DC motor system, the state responses and control effort are shown in Figure 4 and Figure 5. We have run the simulations 100 times. The gray lines in Figure 4 and Figure 5 represent the trajectories of $x_{1}\left(k\right)$, $x_{2}\left(k\right)$, $x_{3}\left(k\right)$, and u(k) for each simulation. The blue line is the average value of ${\left||x\left(k\right)|\right|}^{2}$ and ${\left||u\left(k\right)|\right|}^{2}$ for this 100 simulations which represent their expectation. It can be seen that both the expectation and trajectories converge to zero asymptotically. This verifies the validity of Theorem 2. We can also see that the controller has switched according to a different switching mode. The control effort is a piecewise signal and also finally converges to zero. This implies that the proposed controller can tolerate the mode transmission delay. In order to further show the effectiveness of the proposed method, we take the control gain from reference [11] where no delay and mismatch mode. In this case, $K_{11}={\left[0.2463\;-0.9332\;-5.7399\cdot 10^{-3}\right]}^{T},$ $K_{12}={\left[0.1431\;-0.8488\;-1.9401\cdot 10^{-3}\right]}^{T},$ $K_{13}={\left[0.0911\;-1.5242\;1.7233\cdot 10^{-3}\right]}^{T},$ $K_{21}={\left[0.2463\;-0.9332\;-5.7399\cdot 10^{-3}\right]}^{T},$ $K_{22}={\left[0.1431\;-0.8488\;-1.9401\cdot 10^{-3}\right]}^{T},$ and $K_{23}={\left[0.0911\;-1.5242\;1.7233\cdot 10^{-3}\right]}^{T}$. It can be seen from Figure 6 and Figure 7 that the controller cannot stabilize the considered system.

**Example** **2.** *Consider the system studied in [28], which has three distinct modes. This example can be used to represent some mechatronic system with possible failures in both structure and actuator. The three modes represent that the system suffers from different failures. The initial conditions are selected as x(0)=[1,2] and r(0) is randomly generated from the set M. The system matrices in (1) are given by: $A_{1}=$* [*−0.36 0.69;−1.811.97], $A_{2}=$*[*0.340.62;−0.37 1.36], $A_{3}=$*[*0.340.7;−0.371.36], $B_{1}={\left[-0.1\;0.1\right]}^{T}$, $B_{2}={\left[0.1\;0.1\right]}^{T},$$B_{3}={\left[0\;0.1\right]}^{T}$. We assume that the system dynamics are subjected to a semi-Markov jump process. The transition probability matrix is expressed as Π=[00.40.6;0.500.5;0.70.30]. The sojourn-time PDF $[h_{ij}\left(\tau \right)],\forall i,j\in M$ is given by $h_{11}\left(\tau \right)=h_{22}\left(\tau \right)=h_{33}\left(\tau \right)=0$, $h_{12}\left(\tau \right)=\frac{0.4^{\tau -3}0.6^{7-\tau }4!}{(7-\tau )!(\tau -3)!}$, $h_{13}\left(\tau \right)=\frac{0.3^{\tau -3}0.7^{7-\tau }4!}{(7-\tau )!(\tau -3)!}$, $h_{21}\left(\tau \right)=0.9^{{\left(\tau -3\right)}^{2.2}}-0.9^{{\left(\tau -2\right)}^{2.2}}$, $h_{23}\left(\tau \right)=\frac{0.5^{\tau -3}0.6^{7-\tau }4!}{(7-\tau )!(\tau -3)!}$, $h_{31}\left(\tau \right)=0.4^{{\left(\tau -3\right)}^{1.3}}-0.4^{{\left(\tau -2\right)}^{1.3}}$, and $h_{32}\left(\tau \right)=0.3^{{\left(\tau -3\right)}^{0.9}}-0.3^{{\left(\tau -2\right)}^{0.9}}$. The PDF contain both Weibull and Bernoulli distributions.*

**Figure 8:**
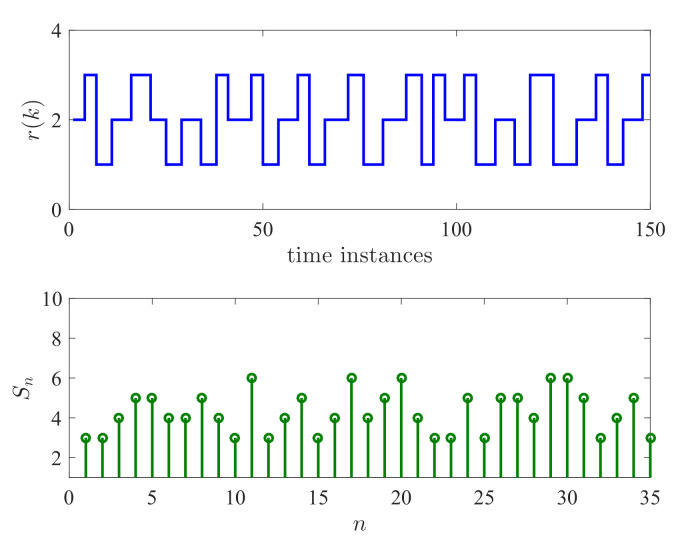
Switching signal r(k) and sojourn time $S_{n}$.

The switching signal r(k) and the sojourn time $S_{n}$ for each mode is shown in Figure 8. It can be seen that both the mode $R_{n}$ and the sojourn time $S_{n}$ are stochastic processes. This implies that r(k) is a semi-Markov jump process.

In order to stabilize the considered system, the asynchronous controller (7) is utilized. Note that Theorem 2 in fact provides a set of LMIs. By solving the LMIs, one can obtain the solution $G_{j}$. Then, the controller can be determined by $K_{j}=K_{j}G_{j}^{-1}.$ We suppose that the controller suffers from a time varying mode transmission delay d(k). Let $\rho_{1}=\rho_{2}=\rho_{3}=2$, ${\underline{\tau }}_{1}={\underline{\tau }}_{2}={\underline{\tau }}_{3}=3$, ${\bar{\tau }}_{1}={\bar{\tau }}_{2}={\bar{\tau }}_{3}=7$, $\tau_{as}=2$ in Theorem 2. Due to $\tau_{as}\leq {\underline{\tau }}_{i}$, (26) and (27) are used for the set $I_{\chi }^{ij}$. Then, we can compute the mode-dependent control gain by solving the LMI in (42)–(47): $K_{1}={\left[7.2934\;-9.9766\right]}^{T},$ $K_{2}={\left[7.5367\;-10.4652\right]}^{T},$ $K_{3}={\left[7.7874\;-10.7313\right]}^{T}.$

**Figure 9:**
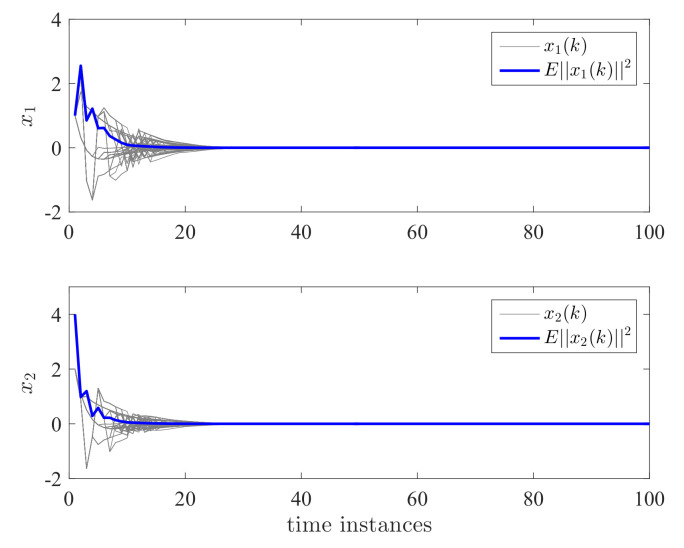
State responses $x_{1}$ and $x_{2}$ for semi-Markov jump systems.

The computed controller is utilized for the DC motor system. In total, 100 simulations have been conducted. As shown in Figure 9, all the trajectories have reached zero, including the state response x(k) for each simulation and ${E||x\left(k\right)||}^{2}$. This shows the effectiveness of the proposed method.

**Example** **3.** *We finally consider a DC system as Example 1 with three distinct modes. The system dynamics is also subjected to a semi-Markov jump process. The transition probability matrix is expressed as Π=[00.40.6;0.500.5;0.70.30]. The sojourn-time PDF $[f_{ij}\left(\tau \right)],\forall i,j\in M$ is given by $h_{11}\left(\tau \right)=h_{22}\left(\tau \right)=h_{33}\left(\tau \right)=0$, $h_{12}\left(\tau \right)=\frac{0.4^{\tau -3}0.6^{10-\tau }7!}{(10-\tau )!(\tau -3)!}$, $h_{13}\left(\tau \right)=\frac{0.3^{\tau -3}0.7^{10-\tau }7!}{(10-\tau )!(\tau -3)!}$, $h_{21}\left(\tau \right)=0.9^{{\left(\tau -1\right)}^{2.2}}-0.9^{\tau {}^{2.2}}$, $h_{23}\left(\tau \right)=\frac{0.5^{\tau -1}0.5^{5-\tau }4!}{(5-\tau )!(\tau -1)!}$, $h_{31}\left(\tau \right)=0.4^{{\left(\tau -3\right)}^{1.3}}-0.4^{{\left(\tau -2\right)}^{1.3}}$, and $h_{32}\left(\tau \right)=0.3^{{\left(\tau -3\right)}^{0.9}}-0.3^{{\left(\tau -2\right)}^{0.9}}$.*

**Figure 10:**
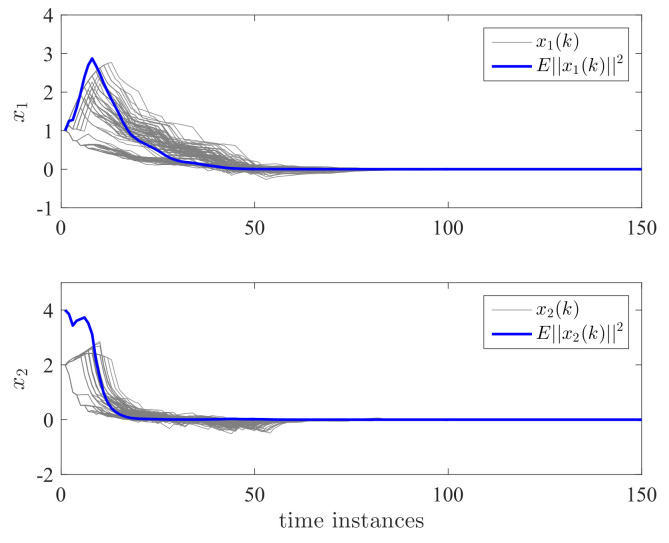
State responses $x_{1}$ and $x_{2}$ for semi-Markov jump systems.

**Figure 11:**
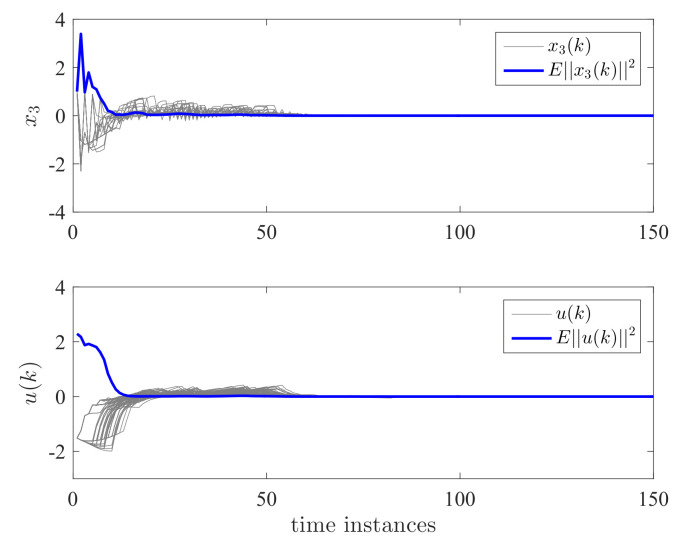
State response $x_{3}$ and control effort *u* for semi-Markov jump systems.

In order to stabilize the DC motor system, the asynchronous controller (7) is adopted. We suppose that the controller suffers from a time constant delay $\tau_{as}=2$. Let $\rho_{1}=\rho_{2}=\rho_{3}=2$, ${\underline{\tau }}_{1}={\underline{\tau }}_{3}=4$, ${\bar{\tau }}_{1}={\bar{\tau }}_{3}=7$, ${\underline{\tau }}_{2}=1$, ${\bar{\tau }}_{2}=5$ in Theorem 2. Due to $\tau_{as}>{\underline{\tau }}_{2}$, (33)–(35) are used for the set $I_{\chi }^{ij}$. Then, the mode-dependent control gain from LMI in (42)–(47) are given by: $K_{1}={\left[0.2493\;-0.8857\;0.0076\right]}^{T},$ $K_{2}={\left[0.2408\;-0.8781\;0.0089\right]}^{T},$ and $K_{3}={\left[0.2446\;-0.8832\;0.0091\right]}^{T}.$ The computed controller is utilized for the DC motor system. It can be seen from Figure 10 and Figure 11 that the state response x(k) in every simulation and ${E||x\left(k\right)||}^{2}$ have all reached zero. This verifies the validity of the proposed controller.

## 5. Conclusions

This paper focuses on the study of asynchronous stabilization of discrete time Markov jump systems. Two classes of typical Markov jump systems are considered, i.e., dual switching systems and semi-Markov jump systems. New stability criteria and numerically testable controller design methods are proposed for these two stochastic switching systems, which can well handle the asynchronous phenomenon. Future works may include extending the proposed results for more complex switched systems. Another interesting research line is considering the control of semi-Markov jump systems under cyber-attacks [41,42]. The additional attacks may further complex the structure of the controller, which is a challenging issue.

## Data Availability

Not applicable.

## Appendix A

### Appendix A.1. Proof of Lemma 1

**Proof.** First, according to the property of Markov process, we have

$$\begin{array}{cc} \; & E\left[V_{g(k+1),r(k+1)}\left(x\left(k+1\right)\right)\right]{|}_{x\left(k\right),\bar{r}(k)=\left[j,j_{1},\ldots ,j_{d(k)-1},i\right]}\\ = & E\left[V_{g(k+1),r(k+1)}\left(x\left(k+1\right)\right)\right]{|}_{\Upsilon (k)} \end{array}$$

where Υ(k)={x(0),r(0),x(1),r(1),…,x(k−d(k)),r(k−d(k)),…,x(k),r(k)}. It follows that

$$\begin{array}{cc} \; & E\left\{E\left[V_{g(k+1),r(k+1)}\left(x\left(k+1\right)\right)\right]{|}_{\Upsilon (k)}\right\}{|}_{x(0),r(0)}\\ = & E\left[V_{g(k+1),r(k+1)}\left(x\left(k+1\right)\right)\right]{|}_{x(0),r(0)} \end{array}$$

where x(0),r(0) are given constants. Therefore, (9) implies that

$$E\left[V_{g(k+1),r(k+1)}\left(x\left(k+1\right)\right)\right]\leq E\left[\chi \left(k\right)V_{g(k),r(k)}\left(x\left(k\right)\right)\right]$$

where ${E\left[\cdot \right]≜E\left[\cdot \right]|}_{x(0),r(0)}$. Then, during the time interval $\left[k_{n},k_{n+1}\right)$, we have

$$\begin{array}{cc} E[V_{g(k),r(k)}\left(x\left(k\right)\right)]\leq & \beta^{|I_{s}|}\alpha^{|I_{as}|}E\left[V_{g\left(k_{n}\right),r\left(k_{n}\right)}\left(x\left(k_{n}\right)\right)\right]\\ \leq & \beta^{k-k_{l}}{\left(\frac{\alpha }{\beta }\right)}^{\tau_{as}}E\left[V_{g\left(k_{n}\right),r\left(k_{n}\right)}\left(x\left(k_{n}\right)\right)\right] \end{array}$$

where $I_{s}$ and $I_{as}$ denote the unions of time intervals that r(k)=r(k−d(k)) and r(k)≠r(k−d(k)) respectively. $|I_{s}|$ and $|I_{as}|$ denote the total lengths of intervals $I_{s}$ and $I_{as}$. Next, by (10)–(11) and (2), we have

$$\begin{array}{cc} \; & E[V_{g(k),r(k)}\left(x\left(k\right)\right)]\\ \leq \; & \beta^{k}{\left(\frac{\alpha }{\beta }\right)}^{N_{\sigma }\left(0,k\right)\tau_{as}}\lambda^{N_{\sigma }\left(0,k\right)}E\left[V_{g(0),r(0)}\left(x\left(0\right)\right)\right].\\ \leq \; & {\left(\frac{\alpha }{\beta }\right)}^{N_{0}\tau_{as}}\lambda^{N_{0}}\cdot e^{(\ln\beta +\frac{\tau_{as}}{\tau_{d}}\ln\left(\frac{\alpha }{\beta }\right)+\frac{\ln\lambda }{\tau_{d}})k}E\left[V_{g(0),r(0)}\left(x\left(0\right)\right)\right]. \end{array}$$

Hence, if (12) holds, we can conclude that $E[V_{g(k),r(k)}\left(x\left(k\right)\right)]\to 0$ as k→+∞. This completes the proof. □

### Appendix A.2. Proof of Lemma 2

**Proof.** (ii)⇒(i). Given a time interval $k\in [k_{n},k_{n+1})$ and consider the following mode-dependent Lyapunov function

$$V_{\nu i}=x^{T}\left(k\right)P_{\nu i}x\left(k\right).$$

As shown in Figure 1, we first consider the case when r(k)≠r(k−d(k)). Hence, we can compute the expectation in (9),

(A1) $$\begin{array}{cc} \; & E\left[\chi \left(k\right)V_{g(k),r(k)}\left(x\left(k\right)\right)\right]{|}_{x\left(k\right),\bar{r}(k)=\left[j,j_{1},\ldots ,j_{d(k)-1},i\right]}\\ =\; & x^{T}\left(k\right)\alpha P_{\nu j}x\left(k\right). \end{array}$$

$$\begin{array}{cc} \; & E\left[V_{g(k+1),r(k+1)}\left(x\left(k+1\right)\right)\right]{|}_{x\left(k\right),\bar{r}(k)=\left[j,j_{1},\ldots ,j_{d(k)-1},i\right]}\\ =\; & E\left[x^{T}\left(k\right)A_{\nu \hat{\nu }j\hat{\varphi }}^{T}P_{\nu ,r(k+1)}A_{\nu \hat{\nu }j\hat{\varphi }}x\left(k\right)\right]{|}_{x\left(k\right),\bar{r}\left(k\right)}. \end{array}$$

Noting the asynchronous behavior described by (5), we have

(A2) $$\begin{array}{cc} \; & E\left[V_{g(k+1),r(k+1)}\left(x\left(k+1\right)\right)\right]{|}_{x\left(k\right),\bar{r}\left(k\right)}\\ =\; & x^{T}\left(k\right)\left(\sum_{\hat{\varphi }=1}^{M_{2}}\mu_{\hat{\nu }i\hat{\varphi }}A_{\nu \hat{\nu }j\hat{\varphi }}^{T}P_{\nu j}A_{\nu \hat{\nu }j\hat{\varphi }}\right)x\left(k\right). \end{array}$$

where $P_{\nu j}=\sum_{l\in M}\pi_{\nu jl}P_{\nu l}$. According to whether or not the bounds of $\pi_{\nu jl}$ are known or not, (A2) can be expressed as

(A3) $$\begin{array}{cc} \; & \sum_{\hat{\varphi }=1}^{M_{2}}\mu_{\hat{\nu }i\hat{\varphi }}A_{\nu \hat{\nu }j\hat{\varphi }}^{T}P_{\nu j}A_{\nu \hat{\nu }j\hat{\varphi }}\\ = & \sum_{\hat{\varphi }\in L}\mu_{\hat{\nu }i\hat{\varphi }}A_{\nu \hat{\nu }j\hat{\varphi }}^{T}\left(\sum_{l\in M_{\nu i}^{K}}\pi_{\nu jl}P_{\nu l}\right)A_{\nu \hat{\nu }j\hat{\varphi }}\\ + & \sum_{\hat{\varphi }\in L}\mu_{\hat{\nu }i\hat{\varphi }}A_{\nu \hat{\nu }j\hat{\varphi }}^{T}\left(\sum_{l\in M_{\nu i}^{UK}}\pi_{\nu jl}P_{\nu l}\right)A_{\nu \hat{\nu }j\hat{\varphi }} \end{array}$$

Note that for $l\in M_{\nu i}^{K}$ and $l\in M_{\nu i}^{UK}$, we have:

$$\begin{array}{cc} \pi_{\nu jl}\leq {\bar{\pi }}_{\nu jl}, & \forall l\in M_{\nu i}^{K} \end{array}$$

$$\begin{array}{cc} \pi_{\nu jl}\leq 1-\sum_{l\in M_{\nu i}^{K}}{\underline{\pi }}_{\nu jl}, & \forall l\in M_{\nu i}^{UK}. \end{array}$$

Hence, we can conclude that

$$\begin{array}{cc} \; & \sum_{\hat{\varphi }=1}^{M_{2}}\mu_{\hat{\nu }i\hat{\varphi }}A_{\nu \hat{\nu }j\hat{\varphi }}^{T}P_{\nu j}A_{\nu \hat{\nu }j\hat{\varphi }}\\ ≺ & \sum_{\hat{\varphi }=1}^{M_{2}}\mu_{\hat{\nu }i\hat{\varphi }}A_{\nu \hat{\nu }j\hat{\varphi }}^{T}P_{\nu j}^{K}A_{\nu \hat{\nu }j\hat{\varphi }}+\sum_{\hat{\varphi }=1}^{M_{2}}\mu_{\hat{\nu }i\hat{\varphi }}A_{\nu \hat{\nu }j\hat{\varphi }}^{T}P_{\nu j}^{UK}A_{\nu \hat{\nu }j\hat{\varphi }} \end{array}$$

It follows that (13)⇒(9) if r(k)≠r(k−d(k)). By the same reasoning, we have (13)⇒(9) if r(k)=r(k−d(k)). Meanwhile, it is obvious that (14)⇒(10). Hence, we show that if statement (*i*) is true, then the system (8) is mean square stable with dwell switching signal g(k) satisfying (12). This completes the proof. (iii)⇔(ii). First note that (A3) is equivalent to the following matrix inequalities.

(A4) $$A_{\nu \hat{\nu }j\hat{\varphi }}^{T}{\tilde{P}}_{\sigma j}A_{\nu \hat{\nu }j\hat{\varphi }}≺T_{\nu \hat{\nu }ij\hat{\varphi }},$$

(A5) $$\sum_{\hat{\varphi }=1}^{M_{2}}\mu_{\hat{\nu }i\hat{\varphi }}T_{\nu \hat{\nu }ij\hat{\varphi }}-{\bar{\chi }}_{\nu \hat{\nu }}P_{\nu j}≺0.$$

where ${\tilde{P}}_{\nu j}=\sum_{l\in M}{\tilde{\pi }}_{\nu jl}P_{\nu l}$. Using the definition ${\tilde{\pi }}_{\nu jl}$ in (18), we have (A4), (A5)⇒(13). For (13)⇒(A4), (A5) since (A3) holds, then there must exists a small constant ε, such that

$$\sum_{\hat{\varphi }=1}^{M_{2}}\mu_{\hat{\nu }i\hat{\varphi }}\left(A_{\nu \hat{\nu }j\hat{\varphi }}^{T}{\tilde{P}}_{\sigma j}A_{\nu \hat{\nu }j\hat{\varphi }}+\varepsilon I\right)-{\bar{\chi }}_{\nu \hat{\nu }}P_{\nu j})≺0.$$

Let $T_{\nu \hat{\nu }ij\hat{\varphi }}=A_{\nu \hat{\nu }j\hat{\varphi }}^{T}{\tilde{P}}_{\sigma j}A_{\nu \hat{\nu }j\hat{\varphi }}+\varepsilon I$, we have (A4), (A5). Next, by using Schur complement, (A4) is equivalent to (16). This completes the proof. □

### Appendix A.3. Proof of Theorem 1

**Proof.** First, performing a congruence transformation to (16) by $\mathrm{diag}\{G_{\hat{\nu }\hat{\varphi }}^{T}\;{\bar{D}}_{\nu }\}$ with ${\bar{D}}_{\nu }≜D_{\nu }^{-1}$ and using the inequalities $-G_{\hat{\nu }\hat{\varphi }}^{T}T_{\nu \hat{\nu }ij\hat{\varphi }}G_{\hat{\nu }\hat{\varphi }}≺T_{\nu \hat{\nu }ij\hat{\varphi }}^{-1}-G_{\hat{\nu }\hat{\varphi }}-G_{\hat{\nu }\hat{\varphi }}$, we can have (19) with ${\bar{T}}_{\nu \hat{\nu }ij\hat{\varphi }}≜T_{\nu \hat{\nu }ij\hat{\varphi }}^{-1}$, $K_{\hat{\nu }\hat{\varphi }}≜K_{\hat{\nu }\hat{\varphi }}G_{\hat{\nu }\hat{\varphi }}.$ Next, performing a congruence transformation to (17) by $Q_{\nu ij\hat{\varphi }}$ and using the inequalities $-Q_{\nu \hat{\nu }ij\hat{\varphi }}P_{\nu j}Q_{\nu \hat{\nu }ij\hat{\varphi }}≺P_{\nu j}^{-1}-2Q_{\nu \hat{\nu }ij\hat{\varphi }}$, we have

(A6) $$\begin{array}{c} Q_{\nu \hat{\nu }ij\hat{\varphi }}T_{\nu \hat{\nu }ij\hat{\varphi }}Q_{\nu \hat{\nu }ij\hat{\varphi }}+{\bar{\chi }}_{\nu \hat{\nu }}P_{\nu j}^{-1}-2{\bar{\chi }}_{\nu \hat{\nu }}Q_{\nu \hat{\nu }ij\hat{\varphi }}≺0. \end{array}$$

Then, by Schur complement and letting ${\bar{P}}_{\nu j}=P_{\nu j}^{-1}$, (A6)⇒(20). This completes the proof. □

### Appendix A.4. Proof of Lemma 3

**Proof.** The proof is similar to the proof of Lemma 1. Note that, we have

$$\begin{array}{cc} \; & E\left[V_{r(k_{n})}\left(x\left(k\right),k-k_{n}\right)\right]{|}_{x\left(k\right),r\left(k_{n}\right)=j,r\left(k_{n-1}\right)=i}\\ =\; & E\left[V_{r(k_{n})}\left(x\left(k\right),k-k_{n}\right)\right]{|}_{\Upsilon (k)} \end{array}$$

$$\begin{array}{cc} \; & E\left\{E\left[V_{r(k_{n})}\left(x\left(k\right),k-k_{n}\right)\right]{|}_{\Upsilon (k)}\right\}{|}_{x(0),r(0)}\\ =\; & E\left[V_{r(k_{n})}\left(x\left(k\right),k-k_{n}\right)\right]{|}_{x(0),r(0)} \end{array}$$

where $\Upsilon \left(k\right)=\{x\left(0\right),r\left(0\right),\ldots ,x\left(k_{n-1}\right),r\left(k_{n-1}\right),x\left(k_{n}\right),r\left(k_{n}\right)\}$. Therefore, from (22) and (23), we can conclude that

$$\begin{array}{cc} \; & E\left[V_{r(k_{n})}\left(x\left(k\right),k-k_{n}\right)\right]{|}_{x(0),r(0)}\\ \leq \; & E\left[\rho_{r(k_{n})}V_{r(k_{n})}\left(x\left(k_{n}\right),0\right)\right]{|}_{x(0),r(0)}\\ \; & k\in N[k_{n}+1,k_{n+1}-1], \end{array}$$

$$\begin{array}{cc} \; & E\left[V_{r(k_{n+1})}\left(x\left(k_{n+1}\right),0\right)\right]{|}_{x(0),r(0)}\\ -\; & E\left[V_{r(k_{n})}\left(x\left(k_{n}\right),0\right)\right]{|}_{x(0),r(0)}\leq K_{3}(||x\left(k\right)||). \end{array}$$

This can complete the proof. □

### Appendix A.5. Proof of Lemma 4

**Proof.** Consider the following mode-dependent Lyapunov function

$$V\left(x\left(k\right),r\left(k\right),\zeta \right)=x^{T}\left(k\right)P_{r(k)}\left(\zeta \right)x\left(k\right)$$

where $\zeta =k-k_{n}$. Meanwhile, let the system mode be $k_{n-1}=i$, $k_{n}=j$, $k_{n+1}=l$ with i,j,l∈M. The proof is divided into the following cases. *Case 1).*d(k) is time invariant and $\tau_{as}>{\underline{\tau }}_{i}.$ In this case, (33)–(35) will be used for set $I_{\chi }^{ij}$. Then, suppose $\zeta \in N\cap [\tau_{as}+1,{\bar{\tau }}_{j}-1].$ From Figure 2, it can be seen that during time interval $\zeta \in [1,\tau_{as}]$, the proposed state feedback controller (7) is asynchronous with the systems mode. Meanwhile, $r(k-\tau_{as})=i$ when $\zeta \in [\tau_{as}-{\underline{\tau }}_{i}+1,\tau_{as}]$. At time interval $\zeta \in [\tau_{as}+1,{\bar{\tau }}_{j}-1]$, the controller is synchronous with the system mode, hence, i.e., $r(k-\tau_{as})=j$. Therefore, the expectation in (22) can be computed as:

$$\begin{array}{cc} \; & E\left[V_{r(k_{n})}\left(x\left(k\right),\zeta \right)\right]{|}_{x\left(k\right),r\left(k_{n}\right)=j,r\left(k_{n-1}\right)=i}\\ =\; & x^{T}\left(k\right)\left({\left(A_{i_{1}j}A_{i_{2}j}\cdots A_{i_{\zeta }j}\right)}^{T}P_{j}\left(\zeta \right)A_{i_{1}j}A_{i_{2}j}\cdots A_{i_{\zeta }j}\right)x\left(k\right), \end{array}$$

$$\begin{array}{cc} \; & E\left[\rho_{r(k_{n})}V_{r(k_{n})}\left(x\left(k_{n}\right),0\right)\right]{|}_{x\left(k\right),r\left(k_{n}\right)=j,r\left(k_{n-1}\right)=i}\\ =\; & x^{T}\left(k\right)\rho_{j}P_{j}\left(0\right)x\left(k\right). \end{array}$$

where $i_{\zeta }≜\left\{i_{1},i_{2},\ldots ,i_{\zeta }\right\}\in I_{\zeta }^{ij}$. Based on the above analysis, (24)⇒(22). Similarly, we have (24)⇒(22) when $\zeta \in [\tau_{as}-{\underline{\tau }}_{i}+1,\tau_{as}]$ and $\zeta \in [1,\tau_{as}-{\underline{\tau }}_{i}]$. In these two situations (34) and (35) will be used for the set $I_{\chi }^{ij}$, respectively. Next, denote the sojourn time $k_{n+1}-k_{n}$ by τ. Similar to the above analysis, we have:

$$\begin{array}{cc} \; & E\left[V_{r(k_{n+1})}\left(x\left(k_{n+1}\right),0\right)\right]{|}_{x\left(k\right),r\left(k_{n}\right)=j,r\left(k_{n-1}\right)=i}\\ =\; & E\left[x^{T}{\left(A_{i_{1}j}\cdots A_{i_{\tau }j}\right)}^{T}P_{l}\left(0\right)A_{i_{1}j}\cdots A_{i_{\tau }j}x\right]{|}_{x\left(k\right),r\left(k_{n}\right),r\left(k_{n-1}\right)}\\ =\; & x^{T}\left(k\right)\sum_{\tau ={\underline{\tau }}_{j}}^{{\bar{\tau }}_{j}}{\left(A_{i_{1}j}A_{i_{2}j}\cdots A_{i_{\tau }j}\right)}^{T}P_{j}\left(\tau \right)A_{i_{1}j}A_{i_{2}j}\cdots A_{i_{\tau }j}x\left(k\right) \end{array}$$

where $i_{\tau }≜\left\{i_{1},i_{2},\ldots ,i_{\tau }\right\}\in I_{\tau }^{ij}$. Note that (33), (34), and (35) will be used for the set $I_{\tau }^{ij}$ when $\tau \in [\tau_{as}+1,{\bar{\tau }}_{j}]$, $\tau \in [\tau_{as}-{\underline{\tau }}_{i}+1,\tau_{as}]$ and $\tau \in [{\underline{\tau }}_{j},\tau_{as}-{\underline{\tau }}_{i}]$ separately. Hence, (25)⇒(23). *Case 2).*d(k) is time invariant and $\tau_{as}\leq {\underline{\tau }}_{i}.$ In this case, (31) and (32) will be used for set $I_{\chi }^{ij}$. Then, suppose $\zeta \in N\cap [\tau_{as}+1,{\bar{\tau }}_{j}-1].$ From Figure 2, the controller is asynchronous with the system mode when $\zeta \in [1,\tau_{as}]$, i.e., $K_{r(k-\tau_{as})}=K_{i}$, and synchronous when $\zeta \in [\tau_{as}+1,{\bar{\tau }}_{j}-1]$, i.e., $K_{r(k-\tau_{as})}=K_{j}$. Therefore, we have

$$\begin{array}{cc} \; & E\left[V_{r(k_{n})}\left(x\left(k\right),\zeta \right)\right]{|}_{x\left(k\right),r\left(k_{n}\right)=j,r\left(k_{n-1}\right)=i}\\ =\; & x^{T}\left(k\right)\left(A_{ij}^{\tau_{as}}{}^{T}\left(A_{jj}^{\zeta -\tau_{as}}\right){}^{T}P_{j}\left(\zeta \right)A_{jj}^{\zeta -\tau_{as}}A_{ij}^{\tau_{as}}\right)x\left(k\right). \end{array}$$

Thus, we can conclude that (24) implies (22). Similarly, we can show (24)⇒(22) when $\zeta \in [1,\tau_{as}]$, and (25)⇒(23). *Case 3).*d(k) is time varying. The proof in this case follows the line of the above two cases. Note that since d(k) is time-varying, then $I_{\chi }^{ij}$ will be defined by (28)–(30) when $\tau_{as}>{\underline{\tau }}_{i},$ and (26)–(27) when $\tau_{as}\leq {\underline{\tau }}_{i}$. □

### Appendix A.6. Proof of Lemma 5

**Proof.** By using Schur complement, (36) and (37) implies

$$A_{i_{p+1}j}{}^{T}\Phi_{j}\left(\zeta ,p+1\right)A_{i_{p+1}j}-\Phi_{j}\left(\zeta ,p\right)≺0,$$

1≤p≤ζ−1;

$$\Phi_{j}\left(\zeta ,0\right)-\rho_{j}\Phi_{j}\left(0,0\right)≺0.$$

where $\forall i_{\zeta }≜\left\{i_{1},i_{2},\ldots ,i_{\zeta }\right\}\in I_{\zeta }^{j}$. It follows that

(A7) $$\left(A_{i_{1}j}\cdots A_{i_{\zeta }j}\right){}^{T}\Phi_{j}\left(\zeta ,\zeta \right)A_{i_{1}j}\cdots A_{i_{\zeta }j}-\rho_{j}\Phi_{j}\left(0,0\right)≺0.$$

By letting $\Phi_{j}\left(\zeta ,\zeta \right)=P_{j}\left(\zeta \right)$, $\Phi_{j}\left(0,0\right)=P_{j}\left(0\right)$, we have (36)–(37)⇒(24). Next, we will show (38)–(41)⇒(25). First note that (25) is equivalent to

(A8) $${\left(A_{i_{1}j}\cdots A_{i_{\tau }j}\right)}^{T}P_{j}\left(\tau \right)A_{i_{1}j}\cdots A_{i_{\tau }j}-T_{ij\tau }≺0,$$

(A9) $$\sum_{\tau ={\underline{\tau }}_{a}}^{{\bar{\tau }}_{a}}T_{ij\tau }-P_{j}\left(0\right)≺0.$$

This can be proved by the same reasoning in the proof of (iii)⇔(ii) in Theorem 2. On the other hand, by Schur complement, (38)–(40) are equivalent to

$$A_{i_{q+1}j}^{T}\Psi_{j}\left(\tau ,q+1\right)A_{i_{q+1}j}-\Psi_{j}\left(\tau ,q\right)≺0,$$

$$\tau_{as}\leq q\leq \tau -1,$$

$$\Psi_{j}\left(\tau ,0\right)-T_{ij\tau }≺0$$

where $\Psi_{j}\left(\tau ,\tau \right)=P_{j}\left(\tau \right)$, $\Phi_{j}\left(0,0\right)=P_{j}\left(0\right)$. It follows that (38)–(40)⇒(A8). Additionally, note that (41)⇔(A9). This shows that (38)–(41)⇒(25). This completes the proof. □

### Appendix A.7. Proof of Theorem 2

**Proof.** First, performing a congruence transformation to (36) by $\mathrm{diag}\{G_{i_{p+1}}^{T}\;{\tilde{\Phi }}_{j}\left(\zeta ,p+1\right)\}$ with ${\tilde{\Phi }}_{j}\left(\zeta ,p+1\right)≜\Phi_{j}^{-1}\left(\zeta ,p+1\right)$ and using the inequalities $-G_{i_{p+1}}^{T}\Phi_{j}\left(\zeta ,p\right)G_{i_{p+1}}≺\Phi_{j}^{-1}\left(\zeta ,p\right)-G_{i_{p+1}}-G_{i_{p+1}}^{T}$, we can have (42) with ${\tilde{\Phi }}_{j}\left(\zeta ,p\right)=\Phi_{j}^{-1}\left(\zeta ,p\right)$, $K_{j}=K_{j}G_{j}.$ Next, we perform a congruence transformation to (38), (39) and (41) by $\mathrm{diag}\{G_{j}^{T}\;\tilde{\Phi }\}$, $\mathrm{diag}\{G_{i_{q+1}}^{T}\;{\tilde{\Psi }}_{j}\left(\tau ,q+1\right)\}$ and $Q_{i\tau }$, respectively, where $\tilde{\Phi }=\Phi^{-1}\left(\zeta ,p\right)$, ${\tilde{\Psi }}_{j}\left(\tau ,q+1\right)=\Psi_{j}\left(\tau ,q+1\right)$. Then, use the inequalities $-G_{j}^{T}\Psi_{j}\left(\tau ,\tau -1\right)G_{j}≺{\tilde{\Psi }}_{j}\left(\tau ,\tau -1\right)-G_{j}-G_{j}^{T}$, $-G_{i_{q+1}}^{T}\Psi_{j}\left(\tau ,q\right)G_{i_{q+1}}≺{\tilde{\Psi }}_{j}\left(\tau ,q\right)-G_{i_{q+1}}-G_{i_{q+1}}^{T}$ and $-Q_{ij\tau }\Phi_{j}\left(0,0\right)Q_{ij\tau }≺{\tilde{\Phi }}_{j}\left(0,0\right)-2Q_{ij\tau }$. We have (44)–(47)⇒(38)–(41). This completes the proof. □
